# Dengue Virus Type 3 Adaptive Changes during Epidemics in São Jose de Rio Preto, Brazil, 2006–2007

**DOI:** 10.1371/journal.pone.0063496

**Published:** 2013-05-07

**Authors:** Christian Julian Villabona-Arenas, Adriano Mondini, Irene Bosch, Diane Schimitt, Carlos E. Calzavara-Silva, Paolo M. de A Zanotto, Maurício L. Nogueira

**Affiliations:** 1 Laboratório de Evolução Molecular e Bioinformática (LEMB), Departamento de Microbiologia, Instituto de Ciências Biomédicas. Universidade de São Paulo, São Paulo, Brazil; 2 Laboratório de Saúde Pública. Departamento de Ciências Biológicas. Faculdade de Ciências Farmacêuticas - Universidade Estadual Paulista “Júlio de Mesquita Filho” Araraquara/SP, Brazil; 3 Division of Heath Science and Technology, Massachusetts Institute of Technology, Cambridge, Massachusetts, United States of America; 4 Department of Infectious Disease and Global Health, Cummings School of Veterinary Medicine, Tufts University, North Grafton, Massachusetts, United States of America; 5 Laboratório de Imunologia Celular e Molecular (LICM), Centro de Pesquisas Rene Rachou (CPqRR), Fundação Oswaldo Cruz (Fiocruz), Belo Horizonte, MG, Brazil; 6 Laboratório de Pesquisas em Virologia, Faculdade de Medicina de São José do Rio Preto, São José do Rio Preto, SP, Brazil; Columbia University, United States of America

## Abstract

Global dengue virus spread in tropical and sub-tropical regions has become a major international public health concern. It is evident that DENV genetic diversity plays a significant role in the immunopathology of the disease and that the identification of polymorphisms associated with adaptive responses is important for vaccine development. The investigation of naturally occurring genomic variants may play an important role in the comprehension of different adaptive strategies used by these mutants to evade the human immune system. In order to elucidate this role we sequenced the complete polyprotein-coding region of thirty-three DENV-3 isolates to characterize variants circulating under high endemicity in the city of São José de Rio Preto, Brazil, during the onset of the 2006-07 epidemic. By inferring the evolutionary history on a local-scale and estimating rates of synonymous (d*S*) and nonsynonimous (d*N*) substitutions, we have documented at least two different introductions of DENV-3 into the city and detected 10 polymorphic codon sites under significant positive selection (d*N*/d*S >* 1) and 8 under significant purifying selection (d*N*/d*S <* 1). We found several polymorphic amino acid coding sites in the envelope (15), NS1 (17), NS2A (11), and NS5 (24) genes, which suggests that these genes may be experiencing relatively recent adaptive changes. Furthermore, some polymorphisms correlated with changes in the immunogenicity of several epitopes. Our study highlights the existence of significant and informative DENV variability at the spatio-temporal scale of an urban outbreak.

## Introduction

Dengue viruses (genus flavivirus, family *Flaviviridae)* are responsible for dengue fever and severe dengue (previously known as dengue hemorrhagic fever, DHF and dengue shock syndrome, DSS), they are some of the most important arthropod-borne viral diseases worldwide [1]–[4]. Increasing incidences of transmission in urban and semi-urban areas in the tropical and sub-tropical countries have promoted dengue to a major public health issue with a hefty economic burden [5]–[10].

Dengue epidemics are largely attributed to three factors: (*i*) Increased urbanization, (*ii*) global dissemination of the major mosquito vectors, *Aedes aegypti* and *Aedes albopictus*, and (*iii*) the interaction and evolution of the four DENV serotypes [11]. Inadequate sustained vector control may partially explain unsuccessful disease prevention in developing countries however variables related to the viruses, the human host and the environment should also be considered [12]. Vaccine development is currently underway as a strategy for controlling this worldwide health threat [13]–[15]. Due to the complexity of DENV dynamics, prevailing serotypes and differences in distribution of viruses within different regions of the globe, serotype and lineage replacement events may play an important role in the evolution of DENV viruses and, consequently must be addressed during vaccine development [16].

Gene genealogy-based DENV evolutionary studies helped characterize the genetic diversity and distribution of different serotypes/genotypes in space and time [17]–[22]. Also, they are important for exploring the role of selection on particular dengue proteins [23]–[29]. Given that serotype-specific neutralizing antibodies confer limited, if any, protection from subsequent infection by the other three serotypes, the appropriate choice of nucleotide residues in the variable genomic regions is critical for the design of effective tetravalent vaccines [30], [31].

Brazil is the South American country with the highest economic impact of dengue and also accounts for the majority of reported cases in the continent as evidenced by a 27 year-long study [8], [32]. The highest dengue incidence was among young adults from 2000 to 2007. However, in 2006 the highest incidence rate of severe dengue increased dramatically among children and remained high during severe epidemics in 2007 and 2008 in the state of Rio de Janeiro [32]–[34]. These circumstances highlight the urgent need for continued studies on dengue molecular epidemiology in order to help determine appropriate policies and effective public health campaigns.

The municipality of São José de Rio Preto (SJRP), one of the 645 municipalities within the State of São Paulo according to the Brazilian Institute of Geography and Statistics (*Instituto Brasileiro de Geografia e Estatística,* IBGE), has a tropical savanna climate (Köppen climate classification), with an annual rainfall of 1410 mm and mean annual temperature of 23.6°C, making it a favorable breeding ground for *Aedes* mosquitoe vectors. Our previous study on dengue in SJRP [35] using a 399 bp-long portion of the NS5 gene, suggested that DENV-3 was introduced into the city in 2005. Since SJRP is one of the municipalities in São Paulo with the highest number of dengue cases reported yearly according to the Epidemiological Surveillance Center of the state (*Centro de Vigilância epidemiológica Alexandre Vranjac, CVE*). We chose to further characterize the isolated DENV-3 strains from SJRP from 2006 to 2007 to gain insights into the viral epidemiology that may help control disease.

## Materials and Methods

### Area of study

The city of SJRP is located in the northwestern region of the state of São Paulo, Brazil (20°49′11″ S and 49°22′46″ W), with a total area of 431 km^2^ and an estimated population of 415,769 inhabitants in 2012 (Data obtained from the IBGE). The infestation by *Aedes* mosquitoes was reported for the first time in 1985 and the first autochthonous cases were reported in 1990, with the introduction of DENV-1 in 1996 (Chiaravalloti-Neto, Superintendence of Endemic Disease Control, SUCEN: personal communication). Unpublished reports by the *Instituto Adolfo Lutz* (Brazilian Public Health Laboratory) point to the introduction of DENV-2 in 1998. Dengue viruses have been endemic in the municipality for almost ten years [36].

### Sequencing genomes

A total of 33 plasma samples were collected from dengue patients during the 2006 and 2007 outbreaks as described elsewhere [35]. Viral RNA was extracted directly from plasma using the QIAamp Viral RNA mini kit (Qiagen). cDNA was synthetized in a 20 µl reverse transcription reaction containing: 1 µl of Superscript III Reverse Transcriptase (Invitrogen), 1 µl of random hexamers (50 ng/µl stock), 1 µL of specific reverse primer 5′AGAACCTGTTGATTCAACAGCAC3′ (10 µM stock) and 5 µl of template RNA. Viral cDNA were diluted to 800 µl in DEPC-treated water and 20 µl were used as template for a 96 specific PCR reactions. Each 10 µl PCR reaction contained: 3 µl of template, 0.03 µl of pfuUltra II polymerase 1 (5 U/µl) (Stratagene), 100 mM dNTPs (Applied Biosystems) and 4 µl of a mixture of forward and reverse primers (0.5 µM stock) (Table S1). Primers were synthesized with M13 sequence tags (forward primers with 5′GTAAAACGACGGCCAGT3′ and reverse primers with 5′CAGGAAACAGCTATGACC3′) so that PCR amplicons could be sequenced using universal M13 forward and reverse primers. Each PCR reaction produced 96 overlapping amplicons, each 500–900 nucleotides in length, which were subsequently sequenced bidirectionally using the Big Dye chemistry on ABI3730xl DNA sequencers (Applied Biosystems). The 33 obtained genomic sequences were submitted to the Broad Institute genome project and deposited in GenBank (Table S2 shows all GenBank accession numbers and name of all samples). (http://www.broadinstitute.org/annotation/viral/Dengue/).

### Phylogenetic Inference

The data set includes the obtained 33 genomic sequences from SJRP that were aligned together with 33 DENV-3 worldwide genomic sequences deposited in GenBank (Table 1) using the Muscle 3.8.31 program [37], [38]. Visual inspection of the alignment and manual editing were done with the Se-Al v2.0 program (http://tree.bio.ed.ac.uk/software/seal/). A Bayesian maximum clade credibility tree was inferred from a set of plausible trees sampled at the stationary phase of four independent Markov Chain Monte Carlo (MCMC) runs with 40 million generations each using a general time reversible (GTR) model of nucleotide substitution [39], a gamma distributed rate variation and a proportion of invariable sites (GTR + **Γ** + I). A relaxed (uncorrelated lognormal) molecular clock [40] and a Bayesian Skyline coalescent tree were used as priors [41]. Analyses were done with Beast v1.6.1 [42]. Convergence of parameters was assessed using Tracer v1.5 program (http://tree.bio.ed.ac.uk/software/tracer/) until all parameters estimates showed Effective Sample Size (ESS) values over 100.

**Table 1:** GenBank and SJRP sequences used for phylogenetic inference.

Country	Year	GenBank Accesion	Genotype
French Polynesia (PF)	1989	AY744678	I
French Polynesia (PF)	1994	AY744685	I
Indonesia (ID)	2004	AY858048	I
Indonesia (ID)	1982	DQ401690	II
Thailand (TH)	1973	DQ863638	II
Thailand (TH)	2001	FJ810414	II
Taiwan (TW)	1998	DQ675531	II
Vietnam (VN)	2006	EU660411	II
Brazil/Acre	2004	EF629367	III
Brazil/Acre	2004	EF629366	III
Brazil/Central West	2001	FJ913015	III
Brazil/Northern	2003	FJ850079	III
Brazil/Northern	2004	FJ850083	III
Brazil/Northern	2005	FJ850086	III
Brazil/Northern	2006	FJ850089	III
Brazil/Northern	2007	FJ850092	III
Brazil/Northern	2008	FJ850094	III
Brazil/Rio de Janeiro	2002	EF629369	III
Brazil/RP	2006	GQ868548	III
Brazil/RP	2006	GQ868547	III
Brazil/RP	2006	GQ868546	III
Brazil	2001	FJ898446	III
Brazil	2001	FJ177308	III
Brazil	2003	FJ898447	III
Colombia (CO)	2007	GQ868578	III
Mozambique (MZ)	1985	FJ882575	III
Nicaragua (NI)	1994	FJ882576	III
Puerto Rico (PR)	2006	EU529692	III
Sri Lanka (LK)	1983	GQ199889	III
Sri Lanka (LK)	1997	GQ252674	III
Trinidad & Tobago (TT)	2002	GQ868617	III
Venezuela (VE)	2007	GQ868587	III
Brazil/Rondonia (BR)	2002	EF629370	V
Brazil/SJRP	2006	RP06161	III
Brazil/SJRP	2006	RP06391	III
Brazil/SJRP	2006	RP06411	III
Brazil/SJRP	2006	RP06611	III
Brazil/SJRP	2006	RP06641	III
Brazil/SJRP	2006	RP06781	III
Brazil/SJRP	2006	RP06801	III
Brazil/SJRP	2006	RP06921	III
Brazil/SJRP	2006	RP06951	III
Brazil/SJRP	2006	RP061901	III
Brazil/SJRP	2006	V065491	III
Brazil/SJRP	2006	RP06171	III
Brazil/SJRP	2006	RP06231	III
Brazil/SJRP	2006	RP06561	III
Brazil/SJRP	2006	RP06651	III
Brazil/SJRP	2006	RP06791	III
Brazil/SJRP	2006	V061231	III
Brazil/SJRP	2006	RP06541	III
Brazil/SJRP	2007	RP07071	III
Brazil/SJRP	2007	RP07111	III
Brazil/SJRP	2007	RP07031	III
Brazil/SJRP	2007	RP07141	III
Brazil/SJRP	2007	RP07181	III
Brazil/SJRP	2007	RP07191	III
Brazil/SJRP	2007	RP07251	III
Brazil/SJRP	2007	RP07371	III
Brazil/SJRP	2007	RP07411	III
Brazil/SJRP	2007	RP07531	III
Brazil/SJRP	2007	RP07601	III
Brazil/SJRP	2007	RP07611	III
Brazil/SJRP	2007	RP07661	III
Brazil/SJRP	2007	RP07751	III
Brazil/SJRP	2007	RP07081	III

### Selection detection analysis

The SJRP dataset was used in a selection analysis to determine whether amino acid positions were subject to negative or positive selection pressures. Comparisons were made with the dengue reference strain Philippines H87/57, following Barrero and Mistchenko [43] general approach. Both genealogy-based, codon-site models *Single Likelihood Ancestor Counting* (SLAC) and the Random-Effects (REL) available in the HyPhy package (www.datamonkey.org) were used to estimate the nonsynonymous (d*N*) and synonymous (d*S*) rates of substitution [44]–[46]. Polymorphisms were analyzed with the program DNASP v5 [47] and subjected to the Tajima D statistic test, as proposed by Tajima [48] to evaluate deviations from the neutral expectation of molecular evolution [49].

### Epitope and immunogenicity prediction

The amino acid sequences coding for the capsid, envelope, NS1, NS2a and NS5 proteins were aligned (http://multalin.toulouse.inra.fr/multalin/multalin.html) and a consensus sequence of each protein was submitted to B (http://www.cbs.dtu.dk/services/BepiPred/) and T (http://www.cbs.dtu.dk/services/NetCTL/) epitope prediction algorithms. The Allele Frequency Net Database (http://www.allelefrequencies.net/) was used to select the HLA classes most predominant in the Southeast of Brazil and used to set up the NetCTL server. The mean value of the epitope propensity scores for each sequence was then classified and plotted according to its potential immunogenicity.

## Results and Discussion

### Complete genome analyzes define distinct DENV3 lineages circulating in SJRP

The availability of high throughput, parallel DNA sequencing allows for comprehensive information on population genetics at the complete viral genome level. In this study, we determined the sequence of 3390 codon-sites of the complete polyprotein from 33 DENV-3 strains isolated during the 2006 and 2007 outbreaks.

**Figure 1:**
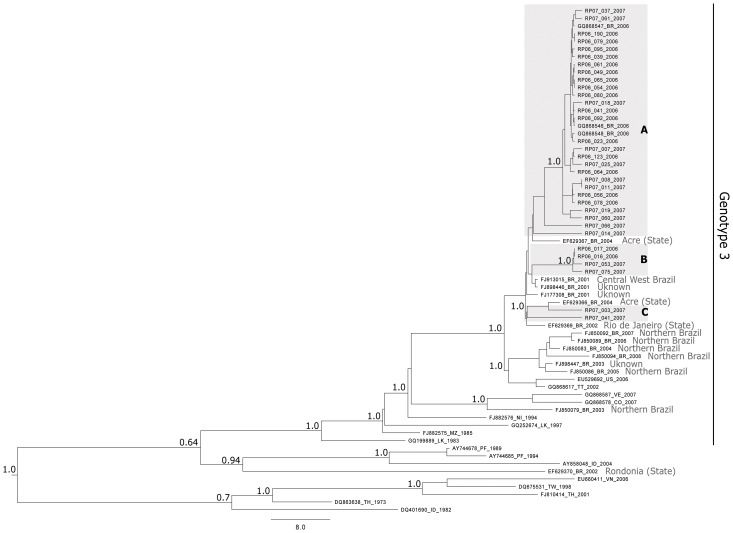
Maximum clade credibility (MMC) tree showing the phylogenetic relationships among SRJP clades (light gray boxes) and other Brazilian isolates. Posterior probability values are shown for main nodes only. Light grey text represents localities (sources) for other Brazilian isolates.

The inferred phylogenetic relationship among DENV-3 strains was summarized in a Bayesian maximum credibility tree (MCC) shown in Figure 1. The majority of Brazilian samples grouped with genotype III, as did most taxa from Latin America isolated since 1994 [20], [50]–[52]. Likewise, the Brazilian strain EF629370 grouped within genotype I, as previously described by Schreiber *et al*. [53]. DENV-3 isolates, other than genotype III, have been reported in Colombia [54] and in the Brazilian states of Minas Gerais and Rondonia, likely imported from Asia [55], [56]. The successive circulation of different genotypes is of public health concern given that distinct combinations could have different epidemic potentials and risk burdens [57].

The samples from SJRP all fell within genotype III. The MCC tree indicates that SJRP sequences group with Brazilian isolates from different regions in highly supported clades (Figure 1). Clade A included most of SJRP isolates and is more closely related to an isolate from Acre in 2004 (EF629367_BR_2004). Clade B includes four SJRP isolates grouping with (*i*) a sample from Central West Brazil (FJ913015_BR_2001) and (*ii*) the Brazilian sample FJ898446_BR_2001 of unknown exactly provenance. Clade C includes two SJRP isolates from 2007 that cluster with a sequence from Rio de Janeiro (EF629369_BR_2002) sampled in 2002. Moreover, in clades A and B there was no temporal separation, suggesting that the 2007 isolates apparently originated from previously circulating 2006 lineages. These results suggest distinct virus introductions into SJRP with one lineage becoming more prevalent and experiencing *in situ* evolution (clade A). Co-circulation of multiple DENV-1 lineages has been documented in Asian and other Brazilian cities [58], [59].

### Succession of DENV-3 genotypes in Brazil

Our data also provides information on the movement of DENV-3 (genotype III) in South America, because sample FJ850079_BR_2002 obtained in the northern part of Brazil grouped with Colombian and Venezuelan sequences suggesting a plausible transmission route.

DENV-3 (genotype III) was isolated for the first time in Brazil from an autochthonous case of DF in December 2000 from Nova Iguaçu, a city located in Rio de Janeiro (RJ) State, which is the second largest metropolitan area in the country, in 2001- 02, a large outbreak of DENV-3 occurred in the neighboring city of Rio de Janeiro [60], [61]. Following its introduction into RJ, the virus spread to the neighboring State of São Paulo (SP), and eventually to the municipality of SJRP. A previous introduction of DENV-3 took place in Brazil in Rondonia State (Amazon region), but sample EF629370_BR_2002 position in the MCC tree indicates it belongs to genotype V with its sister taxa coming from Asia. Nevertheless, most genotype III isolates from the north of Brazil grouped with the Caribbean strains (samples from Puerto Rico and Trinidad and Tobago), which suggests the existence of preferential routes of spread into Brazil, possibly facilitated by anthropogenic factors [51], [62].

### The dynamics of viral change

Sequence comparisons using the reference strain Philippines H87/57 (accession no. M93130) as an outgroup, revealed a total of 893 nucleotide changes, of which 132 corresponded to non-synonymous substitutions. The nucleotide diversity π (that is the average number of nucleotide differences per site between two sequences) [63] within SJRP strains was 0.0052 ± 0.001. Nevertheless, this π value was one order of magnitude lower than that for the complete data set used for phylogenetic inference (n = 0.038 ± 2×10^−5^), which suggests a genetic bottleneck following the introduction into a new human population, with the virus experiencing rapid *in situ* evolution afterwards. This is a likely scenario given that we sampled from the first documented DENV-3 outbreak in a city where the local human population is expected to be largely susceptible to this serotype. Likewise, the average value of Tajimas*' D* for all genes was −1.823 (*p*<0.05) indicating an excess of low frequency polymorphisms, which would be the case in viral populations expanding following a selective sweep.

The Tajima's *D* value near −2 also implies the outcome of negative selection, most likely due to functional constraints imposed on the viral proteins. In agreement with this hypothesis, the overall d*N*/d*S* value for the entire polyprotein coding region was 0.068 (estimated 95% CI from 0.057 to 0.080) that implied purifying (stabilizing) selection. Zanotto *et al*. [64] have argued that at a coarse-grain, the most common pressure acting on DENV is purifying selection with little evidence of recent adaptive evolution when comparing distantly related sequences in space and time, which would be expected due to synonymous change saturation at the serotype level. Moreover, given that arboviruses have adapted to diverse cell types it is generally considered that most mutations produced are likely to be deleterious [24]. Accordingly, the per-site SLAC *dN/dS* analyses revealed that only eight codons were non-neutral and negatively-selected (*p*<0.05) (Table 2). Nevertheless, the presence of low frequency polymorphisms could account for adaptive changes, such as the 10 sites under significant positive selection (Bayes factor >50) that we found with the per-site REL *dN/dS* analyses.

**Table 2:** Non-neutral codons according to the assayed methods (SLAC and REL) at the specified significance levels (p = 0.05 and Bayes factor =  50, respectively).

Region	Codon	Residue	Scaled dN-dS (SLAC)	Bayes Factor (REL)	Selection
Endoplasmic reticulum anchor for the protein C	106	V_24_M_10_		265.737	Positive
Glycoprotein E: central and dimerization domains	355	P_34_	−26.589		Negative
Glycoprotein E: central and dimerization domains	404	P_26_L_7_S_1_		422.066	Positive
Glycoprotein E: central and dimerization domains	449	T_32_I_1_A_1_		310.144	Positive
Glycoprotein E: immunoglobulin-like domain	581	T_33_L_1_		349.007	Positive
Nonstructural NS1	865	D_34_	−35.355		Negative
Nonstructural NS1	893	K_33_L_1_		738.801	Positive
Nonstructural NS1	935	V_34_	−30.491		Negative
Nonstructural NS2B	1401	D_34_	−35.355		Negative
NS3 serine Protease: Domain S7	1634	Y_34_	−35.355		Negative
NS3 serine Protease: ATP binding domain	1829	A_27_V_6_S_1_		80226.4	Positive
Nonstructural NS4A: Cytoplasmic domain	2124	H_34_	−35.355		Negative
Nonstructural NS4A: Lumenal domain	2191	E_29_D_1_A_1_		553.026	Positive
AdoMet-Mtase	2605	P_34_	−30.491		Negative
NS5: Cytoplasmic domain	2862	V_34_	−30.491		Negative
NS5: Cytoplasmic domain	2864	E_20_G_14_		527.608	Positive
NS5: Cytoplasmic domain	2879	K_32_R_2_		548.683	Positive
NS5: Catalytic domain	3129	P_32_L_1_S_1_		286.641	Positive

**Figure 2:**
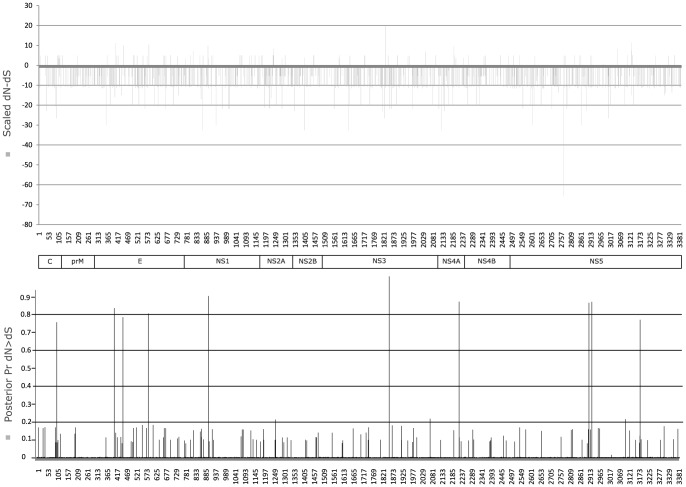
Per-site and per-gene d*N*/d*S* plot. The calculated SLAC d*N*/d*S* and REC posterior probability for positive selection (d*N*>d*S*) are plotted for each site in the upper and lower part of the polyprotein respectively.

Selected sites were located in both structural (S) and nonstructural (NS) proteins (Figure 2). Among the four non-neutral sites within the envelope protein, codon 355 coding for A Proline located in the central and dimerization domains, was under negative selection suggesting its structural importance. Likewise, positively-selected codons 404 and 581 in the envelope are near sites associated with disulfide bonds, suggesting adaptive changes near structurally important residues. Interestingly, Barrero and Mistchenko [43] also identified codons 581 and 893 (within the nonstructural NS1 gene) as positively-selected. King *et al.* [65] detected a statistically significant positively-selected site in the NS1 gene of DENV-3 genotype II. They also detected selective pressure in the C and NS4B genes of genotype I and in the E and NS3 genes of genotype II. We also found additional positively-selected codon sites; 106 (Endoplasmatic reticulum anchor for the protein Capsid), 2191 (Lumenal domain of NS4A) and 3129 (catalytic domain of the RNA-directed RNA polymerase) that fell within domains that are important for membrane interactions/rearrangements and virus replication.

Polymorphism analysis of individual genomic regions indicated that the NS5 and the envelope proteins varied the most with 24 and 22 replacement changes respectively, followed by NS1 (17), NS2A (11), NS4B (8) and Capsid (8) (Table 3). Changes found in the glycoprotein M, the AdoMet-MTase region and the NS3 serine protease were mostly conservative, while replacements in the capsid were less conservative. Twiddy *et al*. [66] found codon sites 169 and 380 to be under positive selection in DENV-3, with the latter being located within the central and dimerization domains of the glycoprotein, which is involved in cell tropism. Likewise, Rodpothong and Auewarakul [25] reported one codon site in the prM gene and twelve residues in the E gene to be under positive selection. In summary, our findings along with others, suggest that genes other than the envelope may experience adaptive changes following introduction into a susceptible population, which implies a possible role for either host-immunity interaction or vector adaptations that require further study.

**Table 3:** Genetic analysis per genomic region.

Region	Codon Position	Sites	Segregating sites	ETA*	Pi**	K***	NS sites	S Sites	TajimaD
Capsid Protein C	13–342	330	26	26	0.009	2.806	8	18	−1.952
Polyprotein Propeptide	355–612	258	18	18	0.005	1.166	3	15	−2.472
Envelope Glycoprotein M	616–840	225	19	19	0.008	1.829	0	19	−2.052
Glycoprotein E: central and dimerization domains	841–1722	882	88	88	0.010	8.513	15	73	−2.257
Glycoprotein E: immunoglobulin-like domain	1726–2013	288	31	31	0.011	3.234	7	24	−2.033
NS1	2323–3387	1065	87	88	0.008	8.011	17	71	−2.344
NS2A	3406–4029	624	66	66	0.010	6.054	11	55	−2.311
NS2B	4039–4419	381	33	34	0.007	2.765	5	29	−2.382
NS3 Serine Protease	4459–4923	465	30	30	0.006	2.749	3	27	−2.211
DEXDc domain	4987–5811	825	59	60	0.008	2.717	8	52	−2.241
NS4A	6286–6720	435	40	40	0.010	4.146	6	34	−2.078
NS4B	6727–7455	729	66	66	0.009	6.496	8	58	−2.210
AdoMet-MTase	7633–8133	501	34	34	0.008	3.972	1	33	−1.864
RNA-directed RNA polymerase	8218–10158	1941	161	162	0.008	15.130	24	138	−2.340
Full coding region	1–10170	10170	888	893	0.008	83.398	132	761	−2.373

* Total number of mutations.

** Nucleotide diversity.

*** Average number of nucleotide differences.

### Short-term evolution of DENV-3 genotype III

Studies documenting selectively driven evolution in DENV are limited and mostly available for serotype 2 [25], [27]–[29], [43], [66], further studies to better characterize possible specific adaptive strategies of each serotype are needed. Therefore we looked at allele replacement in DENV 3 (genotype III).

**Figure 3:**
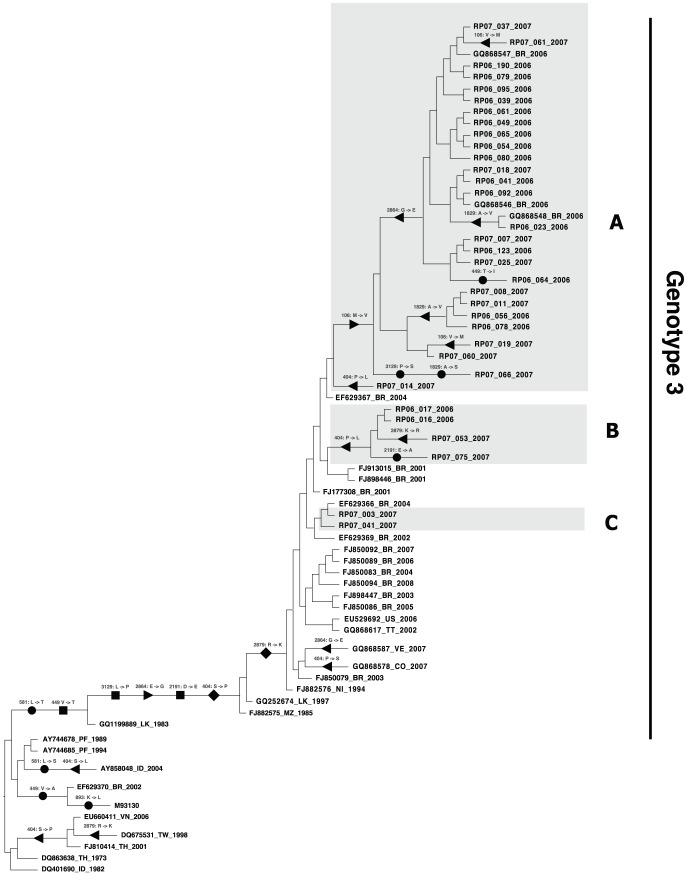
Positively-selected codon transformations tracked over a clade credibility (MMC) tree. Light gray boxes represent SJRP clades. Numbers indicates codon position and one-letter symbols indicate amino-acids. Transformations for each character can be evaluated as follows: • Unique transformation all over the tree; ▪ character state fixed along that branch; ◂ same transformation occurs in another branch (homoplasy); ▸ transformation(s) in the same character occurs along that branch; ⧫ same transformation occurs in another branch (homoplasy) and transformation(s) in the same character occur along that branch.

The allele replacement process, from a population genetics standpoint, has an explicit genealogic representation, since frequency increase of an allele will be manifested by appearing deeper as a synapomorphy in a viral genealogy [67]. Therefore, a synapomorphy indicates a fixed allele in the clade defined by it. To clarify this, we coded each of the ten positively-selected residues as a set of terminal unordered character states, represented as a single capital letter. The most parsimonious reconstruction sets of changes at each internal state in the phylogeny were calculated with MacClade v4.07 [68] using the MCC tree rooted with the reference strain Philippines H87/57. The reconstructed changes are illustrated in Figure 3. They indicate that positively-selected codons (404, 449, 581, 2191, 2879 and 3129) mapped to internal nodes as synapomorphies defining the genotype III. For example, codon 106 Met to Val, (within Protein C, the most non-conservative region in our study) characterized the SJRP clade A and codon 404 Pro to Leu characterized the SJRP clade B. Codon 893 appeared as an autapomorphy for the reference strain H87/57 (M93130). The synapomorphic changes differentiating clades A and B that we observed could reflect some aspects of the intra-genotypic interactions exposed to the human population [22].

Mondini *et al*. [35] described the pattern of spread of two DENV lineages circulating in SJRP, Brazil, during 2006. Based on 82 NS5 sequences, the phylogenetic analysis indicated that all samples were of DENV-3 and related to strains circulating in Martinique during 2000–2001. We have considerably extended that analysis by further showing that different lineages, closely relate to different Brazilian isolates, were co-circulating while experiencing distinct adaptive changes as indicated by their positively-selected synapomorphies. This finding is of relevance, since in several other viral systems, positively-selected changes are adaptive in nature and indicative of adaptive changes imposed by vectors, animal reservoirs or human hosts (*i.e.*, immune escape and herd immunity) [69]–[73].

**Figure 4:**
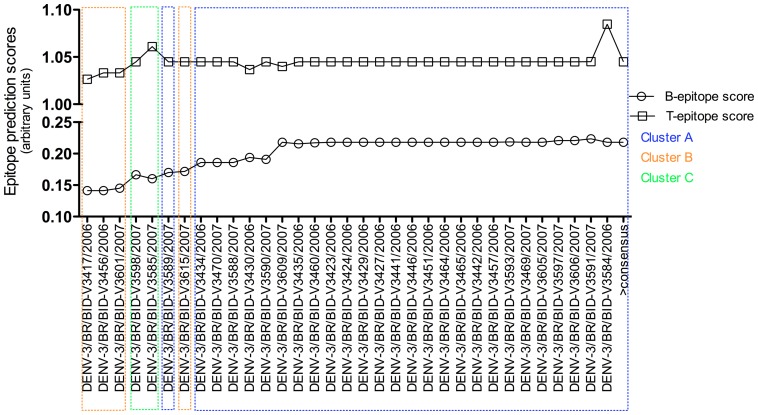
Rank of immunogenicity of DENV-3 sequences. Thirty-three DENV-3 sequences were submitted to B and T epitope prediction using bioinformatics servers and further classified according to their immunogenic potential. Sequences previously clustered in B and C clades have epitopes, in envelope, NS1, NS2a and NS5, putatively less immunogenic when compared to the same epitopes of sequences clustered in clade A, except for sequence ACY70777.1. Substitutions in the capsid were irrelevant.

When we observed that DENV-3 sequences, previously clustered in clades A, B and C (Figure 3), we conducted *in silico* analysis to verify the influence of amino acids substitutions in the immunogenicity of capsid, envelope, NS1, NS2a and NS5 proteins. We found that amino acid substitutions diminished the B/T epitope propensity scores mainly in the DENV-3 sequences clustered in clades B and C (Figure 4). These analyses suggest that amino acid substitutions in sequences grouped in clade A increases its immunogenicity. As a consequence, DENV-3 strains grouped in clade A should expose more antigenic epitopes and more potentially recognized/accessed by the human immune system. We expected that the most immunogenic strains would be negatively selected in a given population. In this particular study we found exactly the opposite. Thus, one can hypothesize that the ADE phenomenon, which would be responsible for an increased pathogenicity and replication in host, can also affect the viral dynamics in a population level. We wonder whether this increased immunogenicity of clade A can induce an antibody dependent enhancement (ADE)-like response in the individual leading to high titers of the virus, which can facilitate the viral transmission to the mosquitoes and, consequently, increase this strain basic reproductive rate. Our findings are of relevance given that ADE may be manifested by challenges imposed by increasing viral immunogenic diversity.

While most studies document the broad geographic expansion of dengue, urban outbreaks dynamics deserves investigation because they contribute to a better understanding of the genetic diversity of strains during local transmission; knowledge that could be exploited for antiviral and vaccine development. In our analyses of 33 complete polyprotein-coding regions we revealed distinct lineages and detected polymorphisms sites that correlated with changes in the immunogenicity of several epitopes. The study provides fine-grain information about the molecular epidemiology of dengue infection.

## Supporting Information

Table S1 **Primers used for amplification and sequencing.**
(DOC)

Table S2 **GenBank accession numbers and name of all samples.**
(XLSX)
