# Correction: Dengue Virus Type 3 Adaptive Changes during Epidemics in São Jose de Rio Preto, Brazil, 2006–2007

**DOI:** 10.1371/annotation/5b2477b7-f68d-4800-a17e-fd85f5ad8311

**Published:** 2013-06-28

**Authors:** Christian Julian Villabona-Arenas, Adriano Mondini, Irene Bosch, Diane Schimitt, Carlos E. Calzavara-Silva, Paolo M. de A Zanotto, Maurício L. Nogueira

The name of the fourth author was spelled incorrectly, and an initial of theirs was also incorrectly omitted. 

The correct name is: Diane J. Schimdt. The correct citation is: Villabona-Arenas CJ, Mondini A, Bosch I, Schimdt DJ, Calzavara-Silva CE, et al. (2013) Dengue Virus Type 3 Adaptive Changes during Epidemics in São Jose de Rio Preto, Brazil, 2006–2007. PLoS ONE 8(5): e63496. doi:10.1371/journal.pone.0063496. 

The correct abbreviation in Contributions is: DJS. 

