# Supplementary material for: Dengue Virus Type 3 Adaptive Changes during Epidemics in São Jose de Rio Preto, Brazil, 2006–2007
Source: PLoS One. 2013 May 7;8(5):e63496. doi: 10.1371/journal.pone.0063496 (PMC3646734; doi:10.1371/journal.pone.0063496)
Supplement: Table S1 — Primers used for amplification and sequencing. (DOC) [file pone.0063496.s001.doc]

**Supplementary Table 1. Primers used for amplification and sequencing**

| **Forward Primer** | **Forward Primer Sequence** | **Reverse Primer** | **Reverse Primer Sequence** |
| --- | --- | --- | --- |
| D3_F_17 | GTAAAACGACGGCCAGTTGGACCGACAAGAACAGTTTC | D3_R_626 | CAGGAAACAGCTATGACCGGTTGCACCAGCAGTCAATG |
| D3_F_17 | GTAAAACGACGGCCAGTTGGACCGACAAGAACAGTTTC | D3_R_797 | CAGGAAACAGCTATGACCCCCATGTCTCTACCTTCTCG |
| D3_Fb_1 | GTAAAACGACGGCCAGTAGTTGTTAGTCTACGTGGACC | D3_R_469 | CAGGAAACAGCTATGACCATTCTTCCCCACAATCATGC |
| D3_F_116 | GTAAAACGACGGCCAGTGACGGGAAAACCGTCTATCA | D3_R_797 | CAGGAAACAGCTATGACCCCCATGTCTCTACCTTCTCG |
| D3_F_1473 | GTAAAACGACGGCCAGTTGGGCTAGAATGYTCACCAC | D3_Rb_2170 | CAGGAAACAGCTATGACCCAGGCTGTGTCTCCCAAGAT |
| D3_F_157 | GTAAAACGACGGCCAGTACCGTGTGTCAACTGGATCA | D3_R_797 | CAGGAAACAGCTATGACCCCCATGTCTCTACCTTCTCG |
| D3_F_238 | GTAAAACGACGGCCAGTTGGCGTTCATAGCTTTCCTCA | D3_R_910 | CAGGAAACAGCTATGACCGGATGGRGTGACCAGCATTA |
| D3_F_1473 | GTAAAACGACGGCCAGTTGGGCTAGAATGYTCACCAC | D3_Rb_2201 | CAGGAAACAGCTATGACCGAATTTARAACACCACCYACTG |
| D3_F_290 | GTAAAACGACGGCCAGTCTTGGCTAGATGGGGAACCT | D3_R_910 | CAGGAAACAGCTATGACCGGATGGRGTGACCAGCATTA |
| D3_F_636 | GTAAAACGACGGCCAGTTGGTGCAACCTYACATCGAC | D3_R_1416 | CAGGAAACAGCTATGACCATGCCTGGGGTGTTATCTCA |
| D3_F_2131 | GTAAAACGACGGCCAGTATGTTCGAGGCCACTGCCA | D3_Rb_2817 | CAGGAAACAGCTATGACCGGTGTGTTTGGCCCRTCTATT |
| D3_F_768 | GTAAAACGACGGCCAGTATGTCGGCTGAAGGAGCTTG | D3_R_1478 | CAGGAAACAGCTATGACCTGTCCGTGGTGARCATTCTA |
| D3_F_2131 | GTAAAACGACGGCCAGTATGTTCGAGGCCACTGCCA | D3_Rb_2862 | CAGGAAACAGCTATGACCCCACCTCCCACACATTCCAT |
| D3_F_798 | GTAAAACGACGGCCAGTGAGAAGGTAGAGACATGGGC | D3_R_1416 | CAGGAAACAGCTATGACCATGCCTGGGGTGTTATCTCA |
| D3_F_2489 | GTAAAACGACGGCCAGTTCCACACCTGGACAGAGCA | D3_Rb_3040 | CAGGAAACAGCTATGACCGATGCYTTTTCTAGCTTCCAACT |
| D3_F_915 | GTAAAACGACGGCCAGTCTGGTCACYCCATCCATGAC | D3_R_1547 | CAGGAAACAGCTATGACCCCATTGYCTATGTACCATCC |
| D3_F_915 | GTAAAACGACGGCCAGTCTGGTCACYCCATCCATGAC | D3_R_1693 | CAGGAAACAGCTATGACCTGCATTGCTCCCTCTTGCGA |
| D3_F_990 | GTAAAACGACGGCCAGTACGTGGGTTGAYGTGGTGCT | D3_R_1787 | CAGGAAACAGCTATGACCCCAATTTGTCCATCTTRAGTC |
| D3_F_1184 | GTAAAACGACGGCCAGTTGAGGAGCAGGACCAGAACTA | D3_R_1798 | CAGGAAACAGCTATGACCCCCTTGAGTTCCAATTTGTC |
| D3_F_1184 | GTAAAACGACGGCCAGTTGAGGAGCAGGACCAGAACTA | D3_R_1816 | CAGGAAACAGCTATGACCCACATTGCATAGCTCATCCC |
| D3_F_1232 | GTAAAACGACGGCCAGTAGGYTGGGGAAACGGTTGTG | D3_R_1948 | CAGGAAACAGCTATGACCGCTTTCCCTTGTCCATCCTC |
| D3_F_1359 | GTAAAACGACGGCCAGTCAGTGCACACAGGAGAYCAA | D3_R_1948 | CAGGAAACAGCTATGACCGCTTTCCCTTGTCCATCCTC |
| D3_F_1473 | GTAAAACGACGGCCAGTTGGGCTAGAATGYTCACCAC | D3_R_1948 | CAGGAAACAGCTATGACCGCTTTCCCTTGTCCATCCTC |
| D3_F_1817 | GTAAAACGACGGCCAGTGGATGAGCTATGCAATGTGCT | D3_R_2491 | CAGGAAACAGCTATGACCTATTGCTCTGTCCAGGTGTG |
| D3_F_1948 | GTAAAACGACGGCCAGTGAGGATGGACAAGGGAAAGC | D3_R_2449 | CAGGAAACAGCTATGACCCCACTTCCACATTTGAGTTC |
| D3_F_1948 | GTAAAACGACGGCCAGTGAGGATGGACAAGGGAAAGC | D3_R_2491 | CAGGAAACAGCTATGACCTATTGCTCTGTCCAGGTGTG |
| D3_F_2038 | GTAAAACGACGGCCAGTGAACCTCCTTTTGGGGAAAG | D3_R_2593 | CAGGAAACAGCTATGACCAGRTTCTCCATTCTGGTTGT |
| D3_F_2131 | GTAAAACGACGGCCAGTATGTTCGAGGCCACTGCCA | D3_R_2879 | CAGGAAACAGCTATGACCTCCRAACCCGTAATCTTCCA |
| D3_F_5792 | GTAAAACGACGGCCAGTCCAAGAAGATGTCTCAAGCC | D3_Rb_6335 | CAGGAAACAGCTATGACCARTCCTTGAATTCCTTGAGYGC |
| D3_F_2587 | GTAAAACGACGGCCAGTAGGTCRACAACCAGAATGGAG | D3_R_3292 | CAGGAAACAGCTATGACCCTCARTGATGGGCCTCTTGT |
| D3_F_2615 | GTAAAACGACGGCCAGTTGTGGAAGCAAATAGCYAATG | D3_R_3292 | CAGGAAACAGCTATGACCCTCARTGATGGGCCTCTTGT |
| D3_F_2756 | GTAAAACGACGGCCAGTCATGGAAAACATGGGGAAAG | D3_R_3292 | CAGGAAACAGCTATGACCCTCARTGATGGGCCTCTTGT |
| D3_F_3026w | GTAAAACGACGGCCAGTGYCAAAAGAATGGWAGTTGG | D3_R_3736w | CAGGAAACAGCTATGACCTYCTYAGGAARAATCCCAAA |
| D3_F_3098 | GTAAAACGACGGCCAGTCACACACTCTYTGGAGCAATG | D3_R_3736w | CAGGAAACAGCTATGACCTYCTYAGGAARAATCCCAAA |
| D3_F_3292 | GTAAAACGACGGCCAGTACAAGAGGCCCATCAYTGAG | D3_R_3954 | CAGGAAACAGCTATGACCTGTTCTCCAGGCAACAGTCA |
| D3_F_3292 | GTAAAACGACGGCCAGTACAAGAGGCCCATCAYTGAG | D3_R_4033w | CAGGAAACAGCTATGACCTTGGRAGCCARTCTGTTTTC |
| D3_F_3390w | GTAAAACGACGGCCAGTAGARGACGGYTGCTGGTATG | D3_R_4033w | CAGGAAACAGCTATGACCTTGGRAGCCARTCTGTTTTC |
| D3_F_3405 | GTAAAACGACGGCCAGTGTATGGCATGGAAATYAGACC | D3_R_4033w | CAGGAAACAGCTATGACCTTGGRAGCCARTCTGTTTTC |
| D3_F_3565w | GTAAAACGACGGCCAGTCATGATTGCRGGGGYTYTCTT | D3_R_4257 | CAGGAAACAGCTATGACCGTCTGCTGACGTGCCAGTTA |
| D3_F_3665 | GTAAAACGACGGCCAGTTCTGACAGRATGGGAATGGG | D3_R_4257 | CAGGAAACAGCTATGACCGTCTGCTGACGTGCCAGTTA |
| D3_F_3665 | GTAAAACGACGGCCAGTTCTGACAGRATGGGAATGGG | D3_R_4487 | CAGGAAACAGCTATGACCTGCTTTTGCCAAGTRTGCCA |
| D3_F_3952 | GTAAAACGACGGCCAGTGTTGACTGTTGCCTGGAGAA | D3_R_4487 | CAGGAAACAGCTATGACCTGCTTTTGCCAAGTRTGCCA |
| D3_F_4250 | GTAAAACGACGGCCAGTTAYGTCATAACTGGCACGTC | D3_Rb_4799 | CAGGAAACAGCTATGACCTCTACRGCAATAACCTGCAC |
| D3_F_4319 | GTAAAACGACGGCCAGTGCTGAGCAAACAGGAGTGTC | D3_R_5099 | CAGGAAACAGCTATGACCGTCTTTCCTGACCCRGGATG |
| D3_F_4351 | GTAAAACGACGGCCAGTGATCACAGTTGATGAYGATGG | D3_Rb_4920 | CAGGAAACAGCTATGACCCTGTTTATGATRGGAGATCCTG |
| D3_F_4661 | GTAAAACGACGGCCAGTATGTGGCACGTCACAAGAGG | D3_R_5409 | CAGGAAACAGCTATGACCCACGAGTTGATATGTACCCT |
| D3_F_4673 | GTAAAACGACGGCCAGTACAAGAGGRGCAGTGTTGAC | D3_R_5409 | CAGGAAACAGCTATGACCCACGAGTTGATATGTACCCT |
| D3_F_4747 | GTAAAACGACGGCCAGTTTCATACGGAGGAGGATGGA | D3_R_5409 | CAGGAAACAGCTATGACCCACGAGTTGATATGTACCCT |
| D3_F_5099 | GTAAAACGACGGCCAGTCATCCYGGGTCAGGAAAGAC | D3_R_5719 | CAGGAAACAGCTATGACCTCACCACRAAGTCCCAATCA |
| D3_F_5099 | GTAAAACGACGGCCAGTCATCCYGGGTCAGGAAAGAC | D3_R_5795 | CAGGAAACAGCTATGACCACTGGCTTGAGACATCTTCT |
| D3_F_5108 | GTAAAACGACGGCCAGTTCAGGAAAGACGCGGAAATA | D3_R_5719 | CAGGAAACAGCTATGACCTCACCACRAAGTCCCAATCA |
| D3_F_5108 | GTAAAACGACGGCCAGTTCAGGAAAGACGCGGAAATA | D3_R_5795 | CAGGAAACAGCTATGACCACTGGCTTGAGACATCTTCT |
| D3_F_5498 | GTAAAACGACGGCCAGTCAGAGCAACGCTCCAATTCAA | D3_R_6122 | CAGGAAACAGCTATGACCTCACCCCTCCTCATGAGTTC |
| D3_F_5544 | GTAAAACGACGGCCAGTGCTCATGGAATTCAGGCAAT | D3_R_6122 | CAGGAAACAGCTATGACCTCACCCCTCCTCATGAGTTC |
| D3_F_5544 | GTAAAACGACGGCCAGTGCTCATGGAATTCAGGCAAT | D3_R_6308 | CAGGAAACAGCTATGACCTCTGAATAAGTGCGGGCATC |
| D3_F_5792 | GTAAAACGACGGCCAGTCCAAGAAGATGTCTCAAGCC | D3_R_6308 | CAGGAAACAGCTATGACCTCTGAATAAGTGCGGGCATC |
| D3_F_6302 | GTAAAACGACGGCCAGTTGGCTTGATGCCCGCACTTA | D3_R_7059 | CAGGAAACAGCTATGACCCGATATYGGCCATCCTTTGT |
| D3_F_6377 | GTAAAACGACGGCCAGTATYGCCCTTGATCTTGTGAC | D3_R_7059 | CAGGAAACAGCTATGACCCGATATYGGCCATCCTTTGT |
| D3_F_6377 | GTAAAACGACGGCCAGTATYGCCCTTGATCTTGTGAC | D3_R_7115 | CAGGAAACAGCTATGACCGGGTTCACTTGTGAATARCA |
| D3_F_6460 | GTAAAACGACGGCCAGTGATGYTGCACACGTCAGAAC | D3_R_7247 | CAGGAAACAGCTATGACCTCCACYGTTGGATTCTTCAT |
| D3_F_6681 | GTAAAACGACGGCCAGTAATGGATCGCGTCGGCYATA | D3_R_7247 | CAGGAAACAGCTATGACCTCCACYGTTGGATTCTTCAT |
| D3_F_5792 | GTAAAACGACGGCCAGTCCAAGAAGATGTCTCAAGCC | D3_Rb_6395 | CAGGAAACAGCTATGACCAGGCACTCTTCCTATYTCTGT |
| D3_F_6776 | GTAAAACGACGGCCAGTGCATATGTCGTGATAGGCAT | D3_R_7428 | CAGGAAACAGCTATGACCTGATCCTTCCCAGAGTGTTG |
| D3_F_6818 | GTAAAACGACGGCCAGTGCAGCCAATGAAATGGGACT | D3_R_7428 | CAGGAAACAGCTATGACCTGATCCTTCCCAGAGTGTTG |
| D3_F_7115 | GTAAAACGACGGCCAGTTGYTATTCACAAGTGAACCCAC | D3_R_7670 | CAGGAAACAGCTATGACCGGCTTCTGTTCTATCCACTTC |
| D3_F_7235 | GTAAAACGACGGCCAGTGCTGCTGGAATAATGAAGAATC | D3_R_7814 | CAGGAAACAGCTATGACCTATGACCAGCCTCCTCTTCC |
| D3_F_7409 | GTAAAACGACGGCCAGTCTAGCCACAGGACCAATAAC | D3_R_7969 | CAGGAAACAGCTATGACCCACACTTTTCAGGTGGAAGA |
| D3_F_7409 | GTAAAACGACGGCCAGTCTAGCCACAGGACCAATAAC | D3_R_8190 | CAGGAAACAGCTATGACCCGTGGAGTTTCGTGAGAGTG |
| D3_F_7540 | GTAAAACGACGGCCAGTCATGAAATCAGTTGGAACAGG | D3_R_8190 | CAGGAAACAGCTATGACCCGTGGAGTTTCGTGAGAGTG |
| D3_F_7540 | GTAAAACGACGGCCAGTCATGAAATCAGTTGGAACAGG | D3_R_8314 | CAGGAAACAGCTATGACCCCACATCTTTCTCTATGGTG |
| D3_F_7784 | GTAAAACGACGGCCAGTCCYGAAGGAAGAGTCATAGA | D3_R_8468 | CAGGAAACAGCTATGACCTCCATGGTAAGCCCACGTT |
| D3_F_8014 | GTAAAACGACGGCCAGTATCYTCACCAAGCCCAACAG | D3_R_8773 | CAGGAAACAGCTATGACCCATAGCYGCGTTGGTTCTGA |
| D3_F_8119 | GTAAAACGACGGCCAGTTACATGCCRACTGTGATTGA | D3_R_8773 | CAGGAAACAGCTATGACCCATAGCYGCGTTGGTTCTGA |
| D3_F_8204 | GTAAAACGACGGCCAGTTCCACGCAYGAAATGTACTGG | D3_R_8902 | CAGGAAACAGCTATGACCTARACGCAGCTTCCACACTT |
| D3_F_8378 | GTAAAACGACGGCCAGTCCCAACATGGAYGTCATTGG | D3_R_8902 | CAGGAAACAGCTATGACCTARACGCAGCTTCCACACTT |
| D3_F_8531 | GTAAAACGACGGCCAGTTGGAGTCGTGAAACTCCTCA | D3_R_9229 | CAGGAAACAGCTATGACCGTTCAGGGTCCATTTGCTGT |
| D3_F_8773w | GTAAAACGACGGCCAGTTCAGAACYAACGCRGCYATG | D3_R_9368w | CAGGAAACAGCTATGACCAAGTYCCYACCTGTCCACT |
| D3_F_8773w | GTAAAACGACGGCCAGTTCAGAACYAACGCRGCYATG | D3_R_9458 | CAGGAAACAGCTATGACCGGTTCTCGAGGTCTGCYTT |
| D3_F_9006 | GTAAAACGACGGCCAGTAGCCAGGTACCTTGAGTTCG | D3_R_9831w | CAGGAAACAGCTATGACCTTGRGCGTAGGCTTTCCCYA |
| D3_F_9202 | GTAAAACGACGGCCAGTTGACCTGCACAATGAGGAAA | D3_R_9913 | CAGGAAACAGCTATGACCCCAATGGRCTGGTACTGCT |
| D3_F_9354 | GTAAAACGACGGCCAGTAAGACCAAAGAGGCAGTGGA | D3_R_9913 | CAGGAAACAGCTATGACCCCAATGGRCTGGTACTGCT |
| D3_F_9369 | GTAAAACGACGGCCAGTGTGGACAGGTGGGRACTTAT | D3_R_10126 | CAGGAAACAGCTATGACCGTTGCTCTGGAAGTGAGAC |
| D3_F_9668 | GTAAAACGACGGCCAGTTGGCATGATTGGCAACAGGT | D3_R_10250 | CAGGAAACAGCTATGACCCAAATGGCTCCCTCTGACTC |
| D3_F_9668 | GTAAAACGACGGCCAGTTGGCATGATTGGCAACAGGT | D3_R_10301 | CAGGAAACAGCTATGACCTGGCTTAARGTGGCCTGACA |
| D3_F_9824 | GTAAAACGACGGCCAGTGCATGYCTAGGGAAAGCCTA | D3_R_10466 | CAGGAAACAGCTATGACCTACACYGTGCGTACAGCTTC |
| D3_F_9824 | GTAAAACGACGGCCAGTGCATGYCTAGGGAAAGCCTA | D3_R_10556 | CAGGAAACAGCTATGACCCTTTGCARGGAGGTACAGC |
| D3_F_10103 | GTAAAACGACGGCCAGTCAATGGTGCGGATCACTCAT | D3_R_10646 | CAGGAAACAGCTATGACCGGAATGATGCTGAGGAGACA |
| D3_F_10103 | GTAAAACGACGGCCAGTCAATGGTGCGGATCACTCAT | D3_R_10668 | CAGGAAACAGCTATGACCCATTTTCTGGCGTTCTGTGC |
| D3_F_5792 | GTAAAACGACGGCCAGTCCAAGAAGATGTCTCAAGCC | D3_Rb_6497 | CAGGAAACAGCTATGACCTARTTCCTCCACYGCATGCCT |
| M13_Forward | GTAAAACGACGGCCAGTAGTTGTTAGTCTACGTGGACC | D3_R_469 | CAGGAAACAGCTATGACCATTCTTCCCCACAATCATGC |
| D3_F_10307 | GTAAAACGACGGCCAGTGGCCACYTTAAGCCACAGTA | M13_Reverse | CAGGAAACAGCTATGACCAGAACCTGTTGATTCAACAGCAC |
| D3_F_6112 | GTAAAACGACGGCCAGTGAACTCATGAGGAGGGGTGA | D3_Rb_6681 | CAGGAAACAGCTATGACCTATRGCCGACGCGATCCATT |
| D3_F_9824 | GTAAAACGACGGCCAGTGCATGYCTAGGGAAAGCCTA | D3_R_10556 | CAGGAAACAGCTATGACCCTTTGCARGGAGGTACAGC |
| D3_F_6302 | GTAAAACGACGGCCAGTTGGCTTGATGCCCGCACTTA | D3_Rb_6776 | CAGGAAACAGCTATGACCGTATGCCTATCACGACATATGC |
| D3_F_10307 | GTAAAACGACGGCCAGTGGCCACYTTAAGCCACAGTA | D3_Rb_10693 | CAGGAAACAGCTATGACCAGAACCTGTTGATTCAACAGCAC |
| D3_F_10307 | GTAAAACGACGGCCAGTGGCCACYTTAAGCCACAGTA | D3_Rb_10693 | CAGGAAACAGCTATGACCAGAACCTGTTGATTCAACAGCAC |
